# Undifferentiated carcinoma of the ampulla of Vater

**DOI:** 10.1186/s40792-016-0284-9

**Published:** 2017-01-06

**Authors:** Misuzu Yamada, Daisuke Furukawa, Naoki Yazawa, Hideki Izumi, Yoshihito Masuoka, Taro Mashiko, Yoshiaki Kawaguchi, Masami Ogawa, Yohei Kawashima, Tetsuya Mine, Kenichi Hirabayashi, Toshio Nakagohri

**Affiliations:** 1 Department of Gastrointestinal Surgery, Tokai University School of Medicine, 143 Shimokasuya, Isehara, Kanagawa 259-1193 Japan; 2 Department of Internal Medicine, Tokai University School of Medicine, 143 Shimokasuya, Isehara, Kanagawa 259-1193 Japan; 3 Department of Pathology, Tokai University School of Medicine, 143 Shimokasuya, Isehara, Kanagawa 259-1193 Japan

**Keywords:** Undifferentiated carcinoma, Ampulla of Vater, Pancreaticoduodenectomy, Gemcitabine, Cisplatin

## Abstract

Undifferentiated carcinoma of the ampulla of Vater is a rare disease with unclear and clinical characteristics and prognosis. Here, we report the case of a 61-year-old man with undifferentiated carcinoma of the ampulla of Vater. He presented to our hospital with an increase in hepatobiliary system enzymes that was detected during a health check-up. Imaging and endoscopy demonstrated a tumor with ulcer in the ampulla of Vater, which was diagnosed as a carcinoma by biopsy. No distant metastasis was observed. Subtotal stomach-preserving pancreaticoduodenectomy was performed. Undifferentiated carcinoma was confirmed based on the presence of small round atypical cells with the formation of a solid alveolar lesion on histopathological examination and immunohistochemical staining that was positive for CAM 5.2 but negative for chromogranin A and synaptophysin. The tumor infiltrated the duodenum, but not the pancreas; no lymph node metastasis was observed. However, liver metastases were detected 2 months postoperatively. Chemotherapy was performed, and the tumor size temporality decreased; however, it grew in size again, and the patients subsequently died of the primary disease 15 months postoperatively. Undifferentiated carcinoma of the ampulla of Vater is a very rare histological type. More number of cases is necessary to clarify optimal treatment.

## Background

Carcinoma of the ampulla of Vater most frequently presents with histopathological findings of cancer of invasive adenocarcinoma accompanied by tubular growth pattern, and undifferentiated carcinoma is rare. In fact, undifferentiated carcinoma is reported to account for 3–7% of gallbladder cancer [1, 2], but only a few cases of extrahepatic bile duct and papillary cancer [3, 4] have been reported. We report the case of a patient with undifferentiated carcinoma that developed in the ampulla of Vater.

## Case presentation

**Fig. 1:**
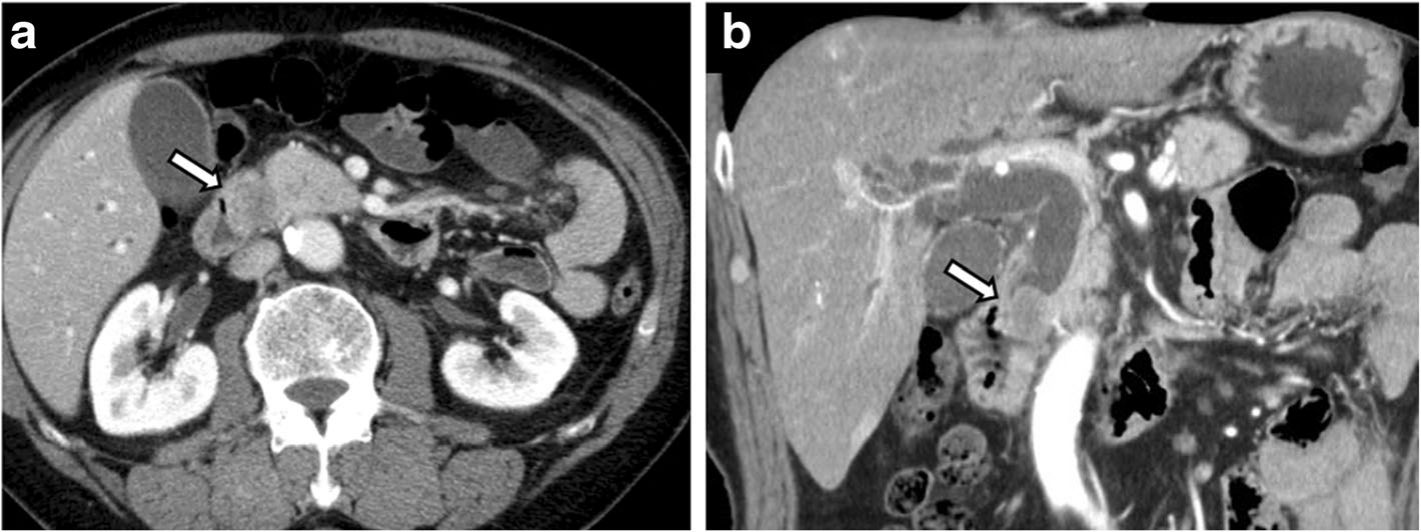
Abdominal CT. Axial (**a**) and coronal (**b**) images show a 3-cm tumor in the ampulla on Vater (*white arrow*) and dilatation of the common bile duct

**Fig. 2:**
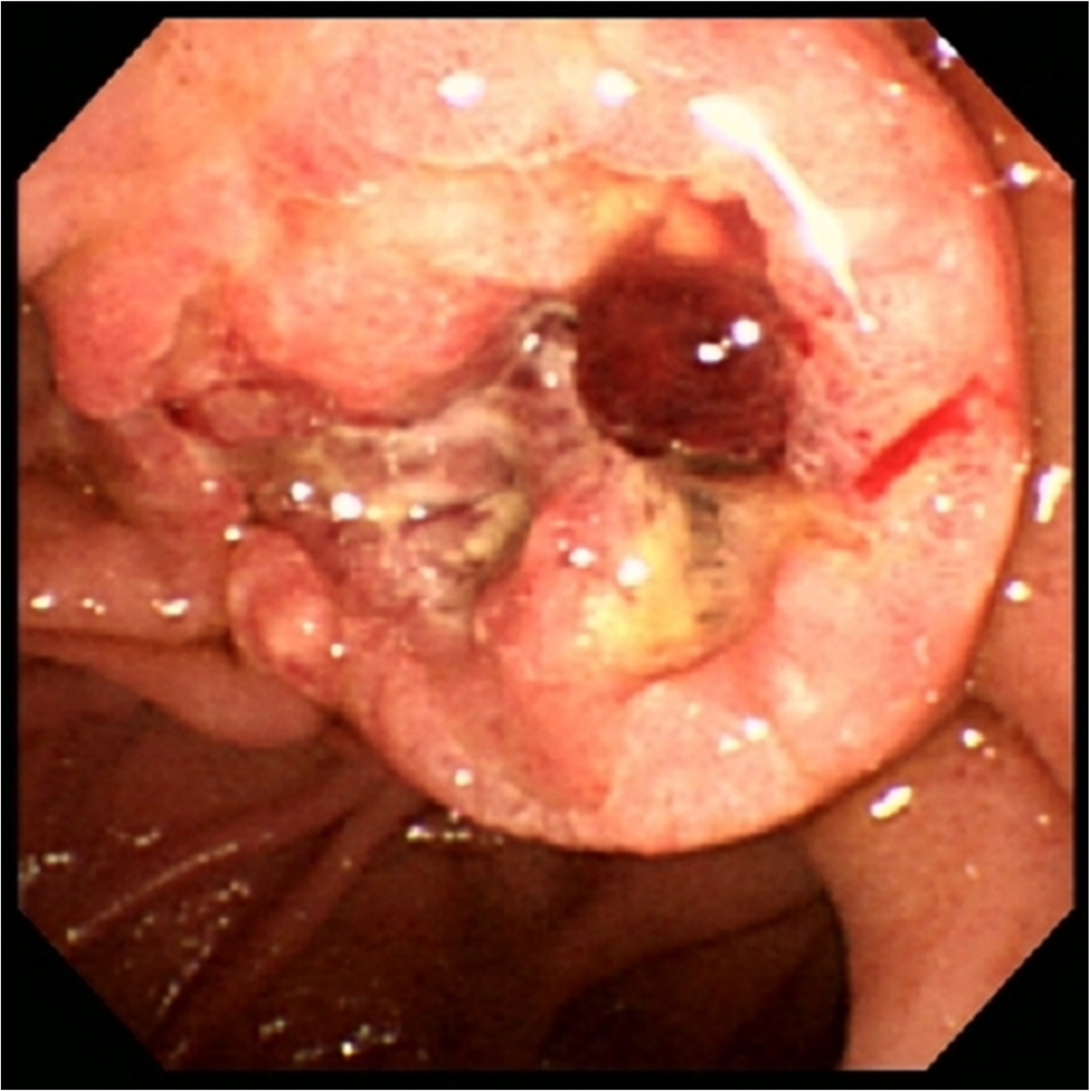
Upper gastrointestinal endoscopy. An ulcerative tumor with raised margins in the ampulla of Vater

The patient was a 61-year-old male who presented to our hospital with increased blood levels of hepatobiliary system enzymes detected during a health check-up. He had a past medical history of hypertension and appendectomy. He smoked ten cigarettes per day for 40 years but had no history of habitual alcohol drinking. There was no pertinent family medical history. His carcinoembryonic antigen level was 9.6 ng/dL, and carbohydrate antigen 19–9 was lower than the detection limit. On abdominal computed tomography (CT), a 3-cm mass was present in the ampullary region, and dilatation of the common and intrahepatic bile ducts were noted (Fig. 1). On upper gastrointestinal endoscopy, an ulcerative tumor with raised margins was observed in the ampulla of Vater (Fig. 2). On histopathological examination of the biopsy specimen, irregular glandular duct structures and small cells were present in a sheet pattern; these findings were suggestive carcinoma, but the histological type could not be identified.

**Fig. 3:**
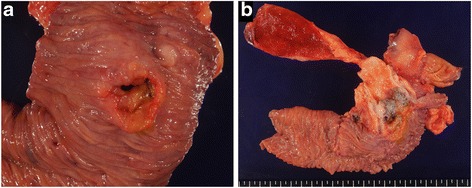
Gross findings of the resected specimen. The resected specimens demonstrated an ulcerative tumor with raised margins (**a**) and involvement of the inferior bile duct (**b**)

**Fig. 4:**
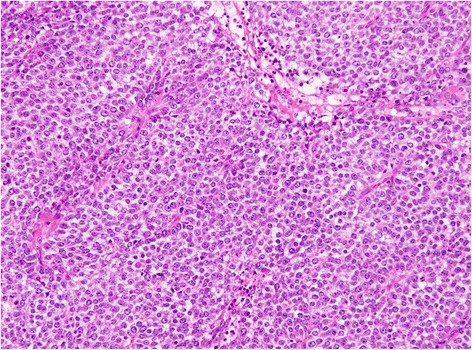
Pathological examination of the resected specimen. The tumor is highly cellular with minimal stroma and small cells with scant cytoplasm arranged in solid nests (Hematoxylin and Eosin stain, ×200)

**Fig. 5:**
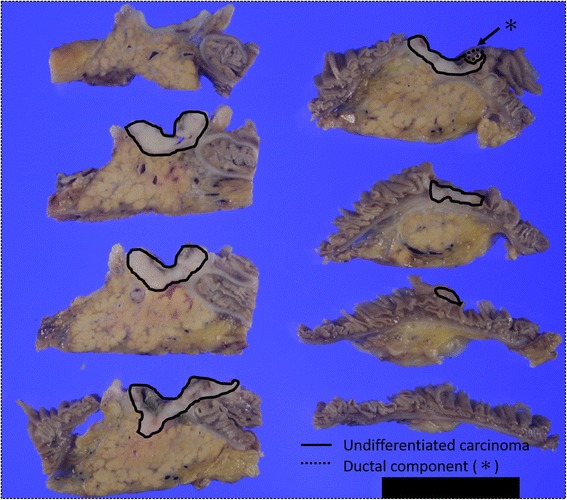
Transversely cut surface of the resected specimen. The small round atypical cells without ductal differentiation are present in the majority of the tumor (*parts surrounded by solid line*). Ductal component is found in the small portion facing the lumen of the duodenum (*part surrounded by dotted line*)

**Fig. 6:**
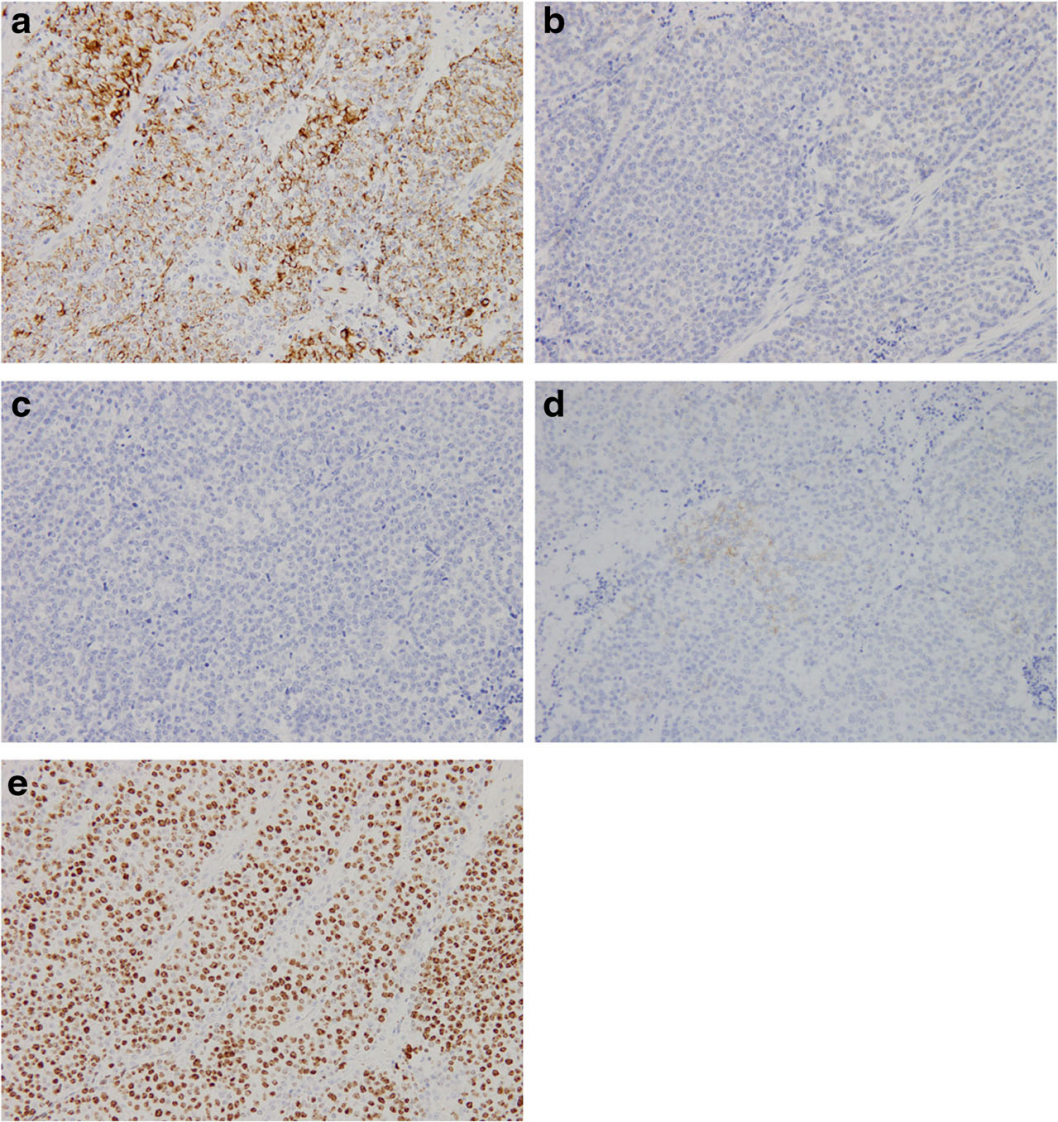
Immunohistochemical staining of the resected specimen. The tumor is positive for CAM 5.2 (**a**) but negative for synaptophysin (**b**) and chromogranin A (**c**). CD56 is weakly positive (**d**). The labeling index of MIB-1 was >90% (**e**) (All photomicrographs, ×200)

Subtotal stomach-preserving pancreaticoduodenectomy was performed (Fig. 3). Histopathological examination of the resected specimen showed small round atypical cells that were mostly forming solid nests without glandular duct structures (Fig. 4). Ductal component was found in the small portion facing the lumen of the duodenum (Fig. 5). No osteoclast-like giant cells or signet-ring cells were noted. On immunohistochemical staining, CAM 5.2 was positive (Fig. 6a), both synaptophysin and chromogranin A were negative (Fig. 6b, c), and CD56 was weakly and focally positive (Fig. 6d). The tumor was finally diagnosed as undifferentiated carcinoma of the ampulla of Vater. The MIB-1 index was higher than 90% (Fig. 6e). The tumor infiltrated the duodenum, but no infiltration in the pancreas was observed. Mild lymphovascular and venous invasion were noted. No perineural invasion or lymph node metastasis was noted. Therefore, this tumor was classified as T2N0M0 (Stage IB). No adjuvant chemotherapy was performed.

**Fig. 7:**
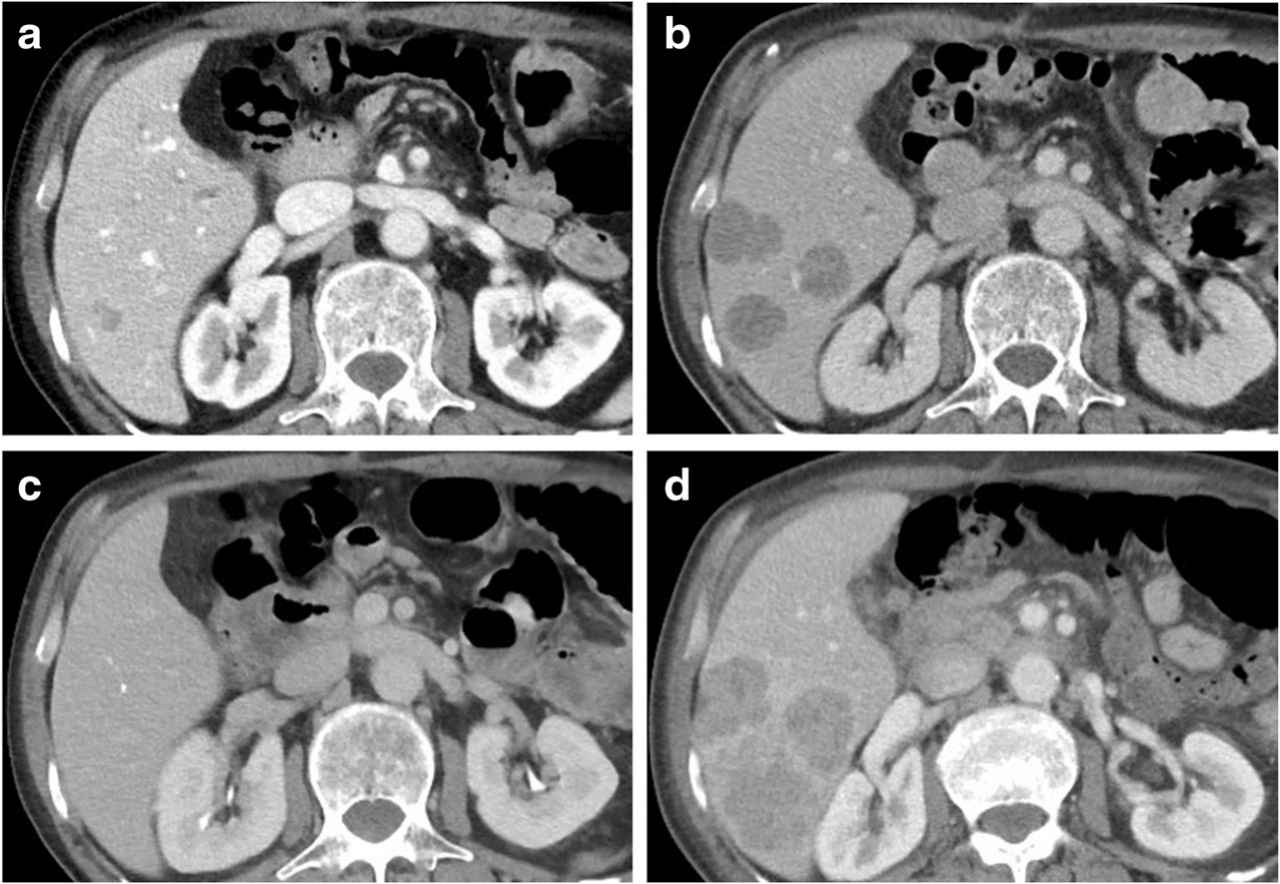
Serial abdominal computed tomography findings. Two months postoperatively, liver metastases are demonstrated (**a**), which increased 6 months postoperatively (**b**). The liver metastases resolved after 2 months of restarting gemcitabine plus cisplatin (**c**), but it enlarged again 2 months later (**d**)

Two months postoperatively, liver metastases were discovered on abdominal CT (Fig. 7a). Combination therapy with gemcitabine plus cisplatin was administered, but the patient developed dyspnea, which was attributed to heart failure secondary to mitral valve prolapse as diagnosed using echocardiography. Considering that the load of cisplatin infusion could aggravate the heart failure, combination chemotherapy was switched to gemcitabine plus S-1. However, the liver metastases were observed to be increased on abdominal CT 6 months postoperatively (Fig. 7b). Therefore, the treatment was switched back to gemcitabine/cisplatin combination therapy with concomitant diuretics. Two months after restarting gemcitabine plus cisplatin, there was resolution of liver metastases as seen on abdominal CT (Fig. 7c). However, 2 month later, abdominal CT revealed that the liver metastases enlarged again despite the continuation of gemcitabine plus cisplatin (Fig. 7d). Moreover, the symptoms of mitral valve prolapse-associated heart failure worsened at this time and continuation of chemotherapy became difficult. The patient died of the primary disease 15 months postoperatively.

### Discussion

This was a rare case of undifferentiated carcinoma of the ampulla of Vater. In this patient, the outcome was poor, as demonstrated by the development of the liver metastases at 2 months and subsequent death at 15 months postoperatively.

According to the World Health Organization classification [5], there are two types of undifferentiated carcinoma of the ampulla of Vater. One has a morphology that is similar to that of neuroendocrine tumor and is formed by relatively small cells with scant cytoplasm, vesicular nuclei, and prominent nuclei arranged in solid sheets or nest. The other is composed of spindle cells and is termed as sarcomatoid carcinoma. Meanwhile, lesions with noticeable osteoclast-like multinucleated giant cells are independently classified as undifferentiated carcinoma with osteoclast-like giant cells. In the present patient, majority of the small atypical cells grew in the medullary portion of the tumor. Chromogranin A, synaptophysin, and CD56 are well-known neuroendocrine markers. Among these, chromogranin A and synaptophysin have been regarded as the most sensitive markers for the diagnosis of neuroendocrine tumors [6]. On the other hand, the specificity of CD56 is questionable [6] and its sensitivity is low [7, 8]. In the present case, CD56 was only weakly and focally positive. Therefore, we did not diagnose neuroendocrine tumor, including small cell carcinoma. There was a ductal component in a small portion of the tumor that faced the lumen of the duodenum; we considered this portion as the site of preoperative biopsy. Although undifferentiated carcinoma is characterized by the absence of glands, the presence of small round atypical cells without ductal differentiation in the majority of the tumor led us to believe that the diagnosis in this case was undifferentiated carcinoma. To the best of our knowledge, there has been only one case report on undifferentiated carcinoma of an ampullary lesion till date in which small cells grew similar to those observed in the present patient [4].

The prognosis of carcinoma of the ampulla of Vater is relatively favorable among the biliary tract carcinoma sites. The overall 5-year survival rate of ampullary carcinoma patients was reported 61–68% [9, 10]. For Stage IB ampullary cancer, which is the same stage as that of our patient, the overall 5-year survival rate has been reported to be 74.7% [9]. Moreover, the median recurrence-free survival was 22.5 months; the incidence of recurrence within 1 year was 21.8% in all patients and 13.8% in patients without lymph node metastasis [11]. Lymph node metastasis and pancreatic invasion were reported to be risk factors for recurrence [12]; these were absent in our patients. However, liver metastases developed 2 months postoperatively, and the outcome was poor compared with other ampullary cancer cases. MIB-1 is a cell growth marker. Although the absence of a relationship between MIB-1 index and prognosis has been reported in ampullary cancer, the high tumor growth ability in the present case, as demonstrated by the high MIB-1 index, may have influenced the poor outcome.

Aside from the ampulla of Vater, the gallbladder and extrahepatic bile duct can also develop undifferentiated biliary tract carcinoma; compared with other histological types, undifferentiated carcinoma of the gallbladder has been reported to have a poor prognosis [2]. On the other hand, undifferentiated carcinoma of the common bile duct grew in a polypoid pattern and could be discovered in an early stage, leading to favorable prognosis [3].

The standard chemotherapy regimen for recurrent biliary tract carcinoma is gemcitabine plus cisplatin [13], but gemcitabine plus S-1 is also effective [14]. In our patient, gemcitabine plus S-1 regimen was ineffective, but the gemcitabine plus cisplatin regimen temporarily reduced the size of liver metastases. Gemcitabine was reported to achieve complete remission of undifferentiated carcinoma with osteoclast-like giant cells in the periampullary region [15]. But the effect of chemotherapy on undifferentiated carcinoma of the biliary tract system is unclear because of the small number of cases. The transient effect observed in the present patient is a matter to be noted and should be taken into account in the future.

## Conclusions

Undifferentiated carcinoma of the ampulla of Vater is a very rare histological type. The present case showed a poor outcome, with development of liver metastases at 2 months and death at 15 months postoperatively. There are very few case reports on undifferentiated carcinoma of the ampulla of Vater, and many points on prognosis and treatment methods remain unclear. More number of cases is necessary to clarify these issues.

## Abbreviations

CT: Computed tomography
